# Editorial: Understanding managers' mental health: the cornerstone for better organizational performance and workers' health

**DOI:** 10.3389/fpsyg.2024.1468825

**Published:** 2024-12-02

**Authors:** Marie-Hélène Gilbert, Julie Dextras-Gauthier, France St-Hilaire, Loïc Lerouge

**Affiliations:** ^1^Département de Management, Université Laval, Québec, QC, Canada; ^2^Département des Sciences de la Santé Communautaire, Université de Sherbrooke, Québec, QC, Canada; ^3^COMPTRASEC (UMR CNRS 5114), CIECST, GPR HOPE Idex, Université de Bordeaux, Bordeaux, France

**Keywords:** managers' mental health, leaders' well-being, leadership, psychosocial risk factors, working conditions, job resources, job demands, coping strategies

Leadership behaviors needed daily to promote teams' performance and employees' mental health have been addressed by numerous studies (e.g., Bakker et al., 2023; Mackay et al., 2022; Montano et al., 2023). While many studies have examined the consequences of managers' behaviors on their employees, very few have looked at the antecedents of those behaviors (e.g., Dextras-Gauthier et al., 2023; Tafvelin et al., 2023). Although managers play a key role in organizations, the contexts in which they operate do not appear to specifically facilitate the adoption of effective leadership behaviors (e.g., Gilbert et al., 2024). Indeed, managers face psychosocial risk factors specific to their functions that may impact their mental health (e.g., Tóth-Király et al., 2024).

The mental health of employees has been widely studied for several decades, while the focus on managers' mental health is quite new in the literature (e.g., Barling and Cloutier, 2017; St-Hilaire and Gilbert, 2019). For this reason, the current discussion on the mental health of managers is timely. Led by an interdisciplinary editorial team, the aim of this Research Topic is to contribute to the advancement of knowledge in this area and deepen our understanding of the factors affecting managers' mental health. Furthermore, this Research Topic provides a better understanding of the conditions under which managers perform their roles and how to better prevent mental health problems among managers.

Two main themes emerge from the papers published on this Research Topic. The first theme explores the work environment in which managers operate. More specifically, it highlights different organizational factors that can positively or negatively affect managers. The second theme relates to the resources and strategies that managers can use to cope with their work environments and to preserve good mental health.

## What is the work environment in which managers navigate?

St-Hilaire and Gilbert (2019) stated, “Being a manager is not necessarily a healthy choice.” They highlighted the many challenges facing managers, including the diversification of the workforce, which implies more complexity; the increase in the number of employees to be supervised; and the many issues related to human resources management. In this Research Topic, Loh and Dollard presented the specific context of upward mistreatment as a constraining context for managers. Based on extensive data collection from managers and employees, the authors highlighted the importance of establishing a psychosocial safety climate as a work environment that is likely to prevent negative behaviors, such as bullying and aggression. In addition, these authors pointed out that upward bullying may occur when employees find themselves in an unfavorable working environment.

One of the main psychosocial constraints identified in the literature are work overload (Campbell and Gavett, 2021). Boucher et al. conducted an exploratory study to gain a better understanding of managers' workload and the factors that influence it. Based on semi-structured interviews with healthcare managers, the authors highlighted the need to examine workload in all its dimensions, in addition to identifying the factors influencing the perceived workload. The authors emphasized that workload can have a negative impact not only on managers' mental health but also on that of the employees they supervise.

Other organizational constraints have also emerged from the literature on managers. For example, Maisonneuve et al. investigated the effects of role ambiguity on first-level healthcare managers. This quantitative study highlighted the mediating role of work addiction in the relationship between role ambiguity and burnout. The authors indicated that role ambiguity prevents managers from investing their resources constructively, which has a negative impact on their mental health.

## What resources and strategies can managers count on?

Gilbert et al. (2024) identified many constraints that managers face daily. The authors stressed the need to reduce managers' constraints so that they can use effectively the resources available to them. In a quantitative study, Sariraei et al. examined the role of emotional intelligence in the relationship between burnout, work motivation, and work behaviors. As past studies (e.g., Kaluza et al., 2020; Parent-Lamarche and Biron, 2022), Sariraei et al. also considered managers' mental health as a predictor of their behaviors. The results of their study, which are nuanced according to three management levels, suggest that emotional intelligence is a resource that is likely to have negative effects on managers' optimal functioning in certain circumstances.

The idea that not all resources are always favorable to the mental health of managers was also discussed in the article by Maisonneuve et al.. The authors examined leader-member exchange quality as a relational resource to better understand the relationship between role ambiguity and burnout. When managers are faced with ambiguity in their roles, they are likely to adopt a strategy of working harder, thus putting themselves at risk of developing work addiction. The higher the leader-member exchange quality, the greater the tendency for managers to invest themselves in work to protect their employees of role ambiguity, which could result in their higher propensity to experience burnout.

Considering that resources to counteract workplace constraints are not always favorable to managers' mental health, they may use different strategies, some of which may have negative effects. Groulx et al.'s article showed that managers can use the strategy of laissez-faire leadership to protect themselves when they feel exhausted during organizational change. From data collected among employees and managers, this study aimed to understand the role of managers' emotional exhaustion on teams' readiness to change by specifically examining the levels of laissez-faire leadership and teams' psychological safety. However, laissez-faire leadership tends to reduce psychological safety, which has a negative impact on teams' readiness to change. Along this line, Boucher et al. also highlighted that although certain strategies used by managers (e.g., extending work hours) may be favorable to mental health in the short term, their use can have negative effects in the longer term.

Finally, the authors contributing to this Research Topic agree on the importance of intervening in managers' mental health at every level of responsibility, whether by implementing a psychosocial safety climate, addressing workloads, or clarifying expectations. The article by Brooks et al. looked specifically at executive coaching as an intervention aimed at reducing burnout symptoms and improving engagement. Based on data collected at two separate points in time, the authors found that a 1-h intervention per week, for 10 weeks, contributed to a reduction in the dimensions of burnout and an increase in managers' vigor compared with those in the control group, who did not participate in the intervention. The different papers of this special issue therefore demonstrate the importance of intervening in organizational constraints so that the resources and strategies managers use do not have deleterious effects. Furthermore, it appears important to implement resources that are specifically aimed at managers and that are consistent with the specific imperatives of their role as managers.

## Conclusion

The papers featured in this Research Topic underscore the difficulties that managers encounter within their professional environments, as well as the complexity of the interactions between the constraints they encounter, the resources they activate, and the strategies they use. What may be considered a resource in one context may act as a constraint in another. In addition, negative managerial behaviors may be strategies aimed at protecting their psychological resources. This Research Topic highlights the importance of deepening the research on managers' mental health to better understand their specific contexts, constraints, resources, and the impact of the strategies managers use in relation to themselves and their teams. It is also important to better understand what leads managers to act in a certain way (i.e., the antecedents of behaviors) to target interventions that are likely to promote their wellbeing and reduce their psychological distress. From a methodological point of view, studies need to be more robust by using longitudinal, multi-level, and multi-source research designs to gain a better understanding of all the factors influencing managers' mental health from different perspectives. Qualitative or mixed research methods also appear particularly relevant to better grasp the contextual elements that influence managers and capture their realities. By combining the efforts of researchers and practitioners, it is possible to generate knowledge that will better promote managers' mental health in organizations, to propose better interventions based on a holistic approach at a collective and individual level, and to enforce legal obligations and duties to foster occupational health in a more optimal and sustainable way.

## References

[B1] Bakker A. B. Hetland J. Olsen O. K. Espevik R. (2023). Daily transformational leadership: a source of inspiration for follower performance? Eur. Manag. J. 41, 700–708. 10.1016/j.emj.2022.04.004

[B2] Barling J. Cloutier A. (2017). Leaders' mental health at work: empirical, methodological, and policy directions. J. Occup. Health Psychol. 22:394. 10.1037/ocp0000055 27732006

[B3] Campbell M. Gavett G. (2021). What covid-19 has done to our well-being, in 12 charts. Har. Bus Review. Available at: https://hbr.org/2021/02/what-covid-19-has-done-to-our-well-being-in-12-charts (accessed February 9, 2022).

[B4] Dextras-Gauthier J. Gilbert M. H. Dima J. Adou L. B. (2023). Organizational culture and leadership behaviors: is manager's psychological health the missing piece? Front. Psychol. 14:1237775. 10.3389/fpsyg.2023.1237775 37842699 PMC10569222

[B5] Gilbert M. H. Dextras-Gauthier J. Boulet M. Auclair I. Dima J. Boucher F.. (2024). Leading well and staying psychologically healthy: the role of resources and constraints for managers in the healthcare sector. J. Health Organ. Manag. 38, 70–91. 10.1108/JHOM-12-2021-0442 38001565

[B6] Kaluza A. J. Boer D. Buengeler C. van Dick R. (2020). Leadership behaviour and leader self-reported well-being: a review, integration and meta-analytic examination. Work Stress 34, 34–56. 10.1080/02678373.2019.1617369

[B7] Mackay M. C. Gilbert M. H. Fournier P. S. Dextras-Gauthier J. Boucher F. (2022). Management behaviors during the COVID-19 pandemic: the case of healthcare middle managers. Front. Psychol. 13:986980. 10.3389/fpsyg.2022.986980 36405185 PMC9670980

[B8] Montano D. Schleu J. E. Hüffmeier J. (2023). A meta-analysis of the relative contribution of leadership styles to followers' mental health. J. Lead. Organ. Stud. 30, 90–107. 10.1177/15480518221114854

[B9] Parent-Lamarche A. Biron C. (2022). When bosses are burned out: psychosocial safety climate and its effect on managerial quality. Int. J. Stress Manag. 29:219. 10.1037/str0000252

[B10] St-Hilaire F. Gilbert M. H. (2019). What do leaders need to know about managers' mental health. Organ. Dyn. 48, 85–92. 10.1016/j.orgdyn.2018.11.002

[B11] Tafvelin S. Lundmark R. von Thiele Schwarz U. Stenling A. (2023). Why do leaders engage in destructive behaviours? The role of leaders' working environment and stress. J. Occup. Organ. Psychol. 96, 165–181. 10.1111/joop.12413

[B12] Tóth-Király I. Katz-Zeitlin E. Houle S. A. Fernet C. Morin A. J. (2024). Managerial leadership behaviors: a longitudinal investigation of the role of job demands and resources, and implications for managers' own well-being. Appl. Psychol. 73, 157–184. 10.1111/apps.12468

